# Privileged Communications

**Published:** 1852-02

**Authors:** 


*Privileged Communications.*—In the October number of this Journal, we spoke of Dr. Storer's address upon medical jurisprudence, quoting some passages from it. The Charleston (S. C.,) Medical Journal and Review noticing the same address, thus speaks:

"Where there is so much to admire, we regret exceedingly that we are forced to take exception to, and enter a protest against the views expressed by Dr. Storer, in reference to the alleged obligation of the physician to reveal secrets that are entrusted to his professional safeguard. For the honor of the profession, we trust that Dr. Storer's opinions have received the concurrence of no one, for, if practically adopted, they would soon bring our noble, our divine calling, into merited disrespect and obloquy.

"Dr. S. holds the following language: 'The most painful duty a medical man is called upon to perform in a Court of Law, if the ends of justice absolutely require it, is to divulge the secrets of his patients, reposed in him in the course of professional confidence. However great may be the struggle within him — however ready and willing he may be to make almost any sacrifice, save that of his integrity, to keep forever locked in his bosom what was sacredly deposited there, the laws of his country are paramount to all other bonds.' And, in support of this opinion, he adduces the charge of Lord Mansfield to Mr. Cæsar Hawkins, in the celebrated trial of the Duchess of Kingston, that he had no privilege to avoid giving evidence, but was bound by the laws of the land to give it, and that he would be clearly justified to the world.

"Dr. S. then says: 'Thus there is no appeal. Those facts must be stated which are necessary to further the ends of justice.'

"Admitting that the laws of some, if not all countries, make it compulsory on physicians to divulge *all* secrets which come to their knowledge, we maintain that the binding force of the *moral*, to preserve them, far transcends that of the *legal* obligation to reveal them. And, let it not be said, that this is a practical application of the political 'higher law' doctrine, at present held by a few fanatics in the United States, for there is not the slightest similitude of the one to the other.

"If Lord Mansfield's opinion be quoted by Dr. Storer, as a guide for the physician, we will place as an offset to it the recent trial of M. Chedanne, at Angers, in France, for refusing to disclose the circumstances that had come to his knowledge, connected with the death of an infant, of which the community had reason from the declaration of M. Chedanne, to suspect, and the autopsy proved, violence as the cause.

"The Superior, which reversed the verdict of the Inferior Court, decided that, although it was *useful* to state every thing relating to the birth and death of a child, yet it was not *essential*. Here, then, is an instance of a high legal tribunal, sustaining the physician in his moral and social obligations! And yet, *perhaps*, this is one of those cases in which the physician commits no breach of moral obligation in divulging the facts as known to him, since it would bring a guilty person — one guilty of infanticide — to punishment. We say, *perhaps*, because the world is not acquainted with the particulars, and M. Chedanne, may have considered himself as morally justified in withholding what he knew."

Having concurred in the opinions expressed by Dr. Storer, we feel ourself somewhat under the censure of our respected contemporary; but we still feel that we are in the right. Not only has this been ruled to be the law by Lord Mansfield, in the trial alluded to, but is the settled ruling of the courts in our own country. As to the case of M. Chedanne, "the world is not acquainted with the particulars," and till it is, it seems premature to quote it. The simple fact is learned from it, that M. Chedanne refused to testify as to certain matters; but till we know more fully the grounds of his refusal, and the opinions expressed both by the Inferior and Superior Courts, the case goes for nothing.

It is, of course, a matter for each individual to decide for himself, when called upon to testify as to such communications, whether or not he will do so; but clearly, such is the law in most of these United States, and the question resolves itself into this, whether or not obedience shall be rendered to the laws. We may say, that we will suffer the penalty; but though we do this, we are still breakers of the law. By no means, in our view, is the judgment of individuals authorized to say, "that the binding force of the *moral* to preserve them, far transcends that of the legal obligation to reveal them." But here we approach a discussion, in which our remarks may be construed to have a bearing upon political topics, entirely foreign to medicine — and we only add, that we apply this principle throughout.

The State of New York has, on this point, advanced somewhat beyond the rest. By statute, it has been enacted that physicians shall be protected as to communications, necessarily made to them, in order that they may be enabled to prescribe.* To our own mind, this seems a wise statute, and we submit it to the profession in our own State, whether or not some exertion should not be made, to procure the enactment of a similar law in New Hampshire. —*New Hampshire Journal of Medicine.*

* The Statute in the State of New York *forbids* the practitioner from revealing, as a witness in Courts of Law, communications derived from professional intercourse with patients. — Editor Buffalo Medical Journal.

